# Symptom clusters and their influencing factors among Vietnamese women after cancer treatment

**DOI:** 10.1038/s41598-025-27892-z

**Published:** 2025-12-04

**Authors:** Huyen Thi Hoa Nguyen, Duc Trung Duong, Tran Ngoc Tran, Anh Chau Nguyen, My Huyen Hac, Quyen Thu Do, Thanh Hai Tran, Duc Quang Tran, Huong Thi Xuan Hoang

**Affiliations:** 1 College of Health Sciences, VinUniversity, Hanoi, Vietnam; 2 Faculty of Health Sciences, Dong Nai Technology University, Bien Hoa, Vietnam; 3 Faculty of Nursing, Phenikaa University, Hanoi, Vietnam

**Keywords:** Symptom clusters, Influencing factors, Cancer, Women, Vietnam, Cancer, Public health, Quality of life

## Abstract

This study aimed to (1) identify symptom clusters in Vietnamese women with cancer and (2) examine the factors influencing those identified clusters. A cross-sectional study was conducted in 5 hospitals across Vietnam from September to December 2023. A total of 217 valid data sets from women with cancer were included. Exploratory factor analysis was applied to identify symptom clusters, followed by structural equation modeling to confirm the underlying structure. Fatigue and appetite loss were recognized as the most common symptoms. The exploratory factor analysis showed two distinct groups of factors, occupying 54.66% of total variance: fatigue, appetite loss, pain, sleep issues, hair loss, nausea, and sexual issues (Factor 1—physical cluster) and mood issues, personal stress, depression, and anxiety (Factor 2—psychological cluster). Multiple linear regression analysis revealed that place of residence (B = 0.318; *p* < 0.05) and occupation (B = 0.263; *p* < 0.05) were significant predictors for the physical cluster. For the psychological cluster, physical activity (B = − 0.599, *p* < 0.001) and the presence of chronic diseases (B = − 0.328, *p* < 0.05) were significant influencing factors, with physical activity demonstrating a strong negative association. The physical and psychological symptom clusters underlie the multidimensional nature of symptom burden in women with cancer and highlight the need for integrative, gender-responsive care models. Culturally tailored, cluster-based interventions are required to enhance survivorship care and patients’ outcomes and quality of life.

## Introduction

### Cancer worldwide and Vietnam

Cancer continues to be a global health challenge, with its incidence rate surpassing 19.9 million cases by 2022^1^. This escalating worldwide burden is influenced by population growth, aging, lifestyle changes, disparities in access to healthcare, and other factors^2^. While cancer impacts are across diverse populations, women bear a disproportionate burden of the disease in prevention, diagnosis, survival rates and supportive care services^3^.

In Southeast Asia, the 2022 diagnosis of over 1 million cancer cases underscores the severity; notably in Vietnam, with breast, lung, and colorectal cancers being predominant among Vietnamese women^1^. Socioeconomic elements, cultural beliefs, and geographic barriers may contribute to delayed care-seeking in cancer outcomes among women in this country^4^. Cancer treatment disrupts lifestyle and mental health, with cultural beliefs and social support guiding coping^5^, family duties and relationships shaping caregivers’ strategies^6^, and religious values influencing how women communicate pain and coping methods^7^. This underscores the importance of gender-specific cancer research and interventions for this particular population, emphasizing the need for increased attention to women’s groups to ensure gender equality in cancer care provision.

### Symptom clusters in cancer

Symptom clusters are the co-occurrence of two or more interrelated symptoms that may share common mechanisms^8–10^, with a sentinel symptom playing a key role in predicting related symptoms^11^, and the cluster’s stability is defined by its enduring nature and symptom consistency^12^. For instance, heightened fatigue levels may exacerbate pain, sleep disturbances, and emotional distress within a cluster^11^. Exploring these “symptom clusters” provides insights for unified interventions, conserving resources, and mitigating healthcare costs while elevating the overall quality of care and life for affected individuals^12^. Given that symptoms of cancer are experienced differently by males and females^13,14^, it is essential to examine symptom clusters through a gender-specific lens. The variability of measurement tools for assessing symptom clusters has introduced inconsistencies across studies, emphasizing the need for further research to facilitate a systematic review and the development of a unified symptom cluster framework.

### Conceptual framework: factors influencing symptom clusters

Multiple interrelated factors shape symptom cluster experiences in women cancer care, encompassing demographic, clinical, behavioral and psychological dimensions. Demographic and socioeconomic factors significantly influence susceptibility, with younger age, unmarried/unpartnered status, unemployment, lower income, and lower educational attainment correlating with heightened symptom burden across cancer types^15–17^. Clinical characteristics, including cancer diagnosis, advanced tumor stage, metastatic disease, and treatment modalities (chemotherapy frequently causing gastrointestinal-nutrition clusters, while radiation often associates with fatigue-skin toxicity clusters), dictate cluster composition and trajectory^15,16,18^. Lifestyle factors such as regular physical activity exerts anti-inflammatory effects by reducing pro-inflammatory cytokines (e.g., IL-6, TNF-α) and modulating insulin resistance, thereby mitigating fatigue-depression-pain clusters commonly observed in breast and gynecological cancers^19,20^. Psychological factors are integral, as anxiety and depression frequently co-occur within clusters and demonstrate bidirectional relationships with physical symptoms, amplifying overall distress and impairing self-efficacy and self-care capacity^18,21^. Based on this evidence, the multivariate models will include independent variables from these four domains to determine their unique associations with the symptom-cluster scores.

In Vietnam, the escalating cancer incidence witnessed a threefold increase over the past 30 years, which accentuates the urgency to explore symptom clusters comprehensively^22^. In 2018, breast and cervical cancers, specifically affecting women, accounted for a total of 11.7% of cancer cases while the total cancer incidence was 164,671 in Vietnam^23^. Despite the pervasive nature of interconnected symptoms or symptom clusters in cancer patients, Vietnam’s symptom research has predominantly prioritized evaluating and mitigating individual symptoms^22,24^. Moreover, there is a shortage of studies addressing symptom clusters among Vietnamese women with cancer. Our study thus assumes a pivotal role in bridging this knowledge gap, aiming to contribute meaningful insights to the existing body of research and data pool.

Our primary objectives are to:

1. Identify symptom clusters in women with cancer in Vietnam; and
2. Analyze the factors influencing identified symptom clusters.

This research not only sheds light on the specific challenges faced by Vietnamese women with cancer-related symptoms but also lays the groundwork for targeted interventions. By doing so, our findings aim to inform tailored interventions, ultimately easing the overall quality of life and treatment outcomes for women dealing with cancer in Vietnam’s unique context.

## Methods

### Sample and setting

A cross-sectional study was conducted with a convenience sample of women with cancer in 5 hospitals across Vietnam from September to December 2023. The inclusion criteria for women included (1) Vietnamese women who able to speak and write in Vietnamese and living in Vietnam at the time participating in the project, (2) > 18 years–old, (3) be diagnosed with at least one type of cancer or have been finished cancer treatment, and (4) willingness to participate in this study. The study excluded those who are diagnosed with mental illness.

The sample size was calculated (just used for target population) based on the formula N ≥ 104 + m (m: number of independent variables). There are 10 independent variables; therefore, the minimum required sample size is 114^25^. In total, we collected 318 cancer women who were eligible and participated in the study. After data cleaning with removing incomplete questionnaires, inconsistent provided information, and duplicate entries, we obtained a total of 217 participants. Given that Structural Equation Modeling (SEM) was used in the analysis, the adequacy of the sample size was also evaluated. Following Hair et al. (2019), an estimation of 10–20 observations per parameter is recommended for SEM to maintain power of analysis^26^. Our model includes approximately 12 parameters, which would require at least 120–240 participants. With a final sample size of 217, our study met the recommended threshold for SEM analysis.

### Measures

#### Demographic characteristics

Demographic information included age, gender, living area, religion, level of education, employment, personal income per month, and family member support, and also patient health history related to cancer. In addition, some behavioral and lifestyle characteristics were also asked such as smoking, physical activities and type of exercise, sleeping disturbances, and strategies used to monitor and manage symptoms. In particular, physical activity means if participants engaged in at least 30 min of moderate-intensity exercise three or more times per week^27^.

#### Assessing symptoms

The number of physical and psychological symptoms were collected including pain, fatigue, insomnia, nausea/vomiting, appetite loss, hair loss, sexual issue, mood issues^28^. Symptoms are reflected with the frequency from “never” to “always”. The score of each statement is evaluated on a 5-level Likert scale, with 0-never, 1-rarely, 2-occasionally, 3-sometimes, 4-usually, 5-always. The properties of instruments were reported in the previous study with Cronbach’s alpha of 0.85—a strong internal consistency. Inter-item correlations ranged from 0.21 to 0.80, mostly within the acceptable range.

#### Verbal pain scale (VPS)

Participants’ pain levels were evaluated using the Verbal Pain Scale, a popular pain rating scale that is available in 13 different languages, including Vietnamese^29^. The VPS has a 10-point scale ranging from 0 to 10, with higher scores indicating higher pain levels.

#### Karnofsky performance status scale

The Karnofsky Performance Status Scale was used widely to measure the level of functional capacity in patients living with cancer. The KPS has been used in Vietnamese clinical settings for oncology patients^22,30^. This scale rates the level of functional capacity on a scale from 20 to 100%, with higher percentages indicating better functional performance status.

#### Mental health (PHQ-9, GAD-7, and PSS-10)

Patient Health Questionnaire (PHQ-9) was used to screen for depression in women with cancer^31,32^. This instrument can assess depressive symptoms and suggest grade depressive symptom severity and has been indicated a Cronbach’s alpha of 0.70 to 0.80^33,34^. Levels of depression severity is rated according to PHQ-9 score with minimal level (grade from 0 to 4), Mild level (from 5 to 9), Moderate level (from 10 to 14), moderately severe (from 15 to 19), and severe level of depression (from 20 to 27).

General Anxiety Disorder (GAD-7) was used to assess anxiety^35^. The GAD-7 has been validated in Vietnam with Cronbach alpha of 0.91^33^. Seven items in GAD-7 designed to self-report the anxiety of an individual during the previous two weeks is rated from 0 to 3, corresponding to “not at all,” “several days,” “more than half the day,” and “nearly every day,” respectively. The total score is summed up and range from 0 to 21. The cut-off points for mild, moderate, and severe anxiety are represented as 5, 10, and 15, respectively.

The Vietnamese version of the Perceived Stress Scale (PSS-10) was used to assess the self-reported stress among participants (Cronbach’s alpha of 0.80)^36^. Participants were asked how often they experience thoughts and feelings during the last months through 10 items. Each item is responded from 0 (never) to 4 (very often). The scores is aggregated, and the possible total score ranges from 0 to 40. Higher total scores show a higher likelihood that environmental demands exceed the ability to cope in individuals.

### Procedures

The study was approved by the Scientific Council, Ethics Council in Biomedical Research, Vinmec International General Hospital (No.75/2022/QD-VMEC dated July 26, 2022). The researchers obtained a list of women with cancer who had received treatment at the selected hospitals, along with their contact information (usually phone numbers). Eligible participants were invited to participate in the study through phone calls or in-person invitations at the hospital. Participants who agreed to participate and met the inclusion criteria were provided with an information sheet about the study and a consent form. After obtaining written consent, participants completed the survey questionnaire in the presence of the investigator. The data collection process took approximately 15 min per participant. If doubts arose regarding the interpretation of the instructions, the investigator would assist them. After the questionnaire was completed, it was immediately collected by the administering investigator.

### Statistical analysis

Data analyses were conducted using IBM SPSS version 26.0 and AMOS version 20.0. Demographic information, clinical characteristics, and symptom incidence and severity were analyzed using descriptive statistical methods. Exploratory factor analysis was used to extract symptom clusters, and univariate, bivariate analyses and multiple linear regression analyses were performed to explore factors affecting symptom clusters. Independent variables included demographic, clinical, and behavioral characteristics (e.g., age, residence, occupation, physical activity, presence of chronic diseases), while the dependent variables were the scores of the physical and psychological symptom clusters. Multiple linear regression analyses were first conducted to explore associations between demographic, clinical, and behavioral variables and symptom clusters. These analyses served as a preliminary step to identify potential influencing factors. Structural equation modeling (SEM) using AMOS version 20.0 with maximum likelihood estimation was subsequently used as a confirmatory method to test the hypothesized relationships and examine the model structure, accounting for measurement errors and latent variables. The criteria used to appraise the structural model were model fit indices, as well as the magnitude and direction of path estimates^26^. The fit indices that were used to evaluate the proposed model were normed Chi-square (χ2/df), Goodness-of-Fit Index (GFI), Adjusted Goodness-of-Fit Index (AGFI), Comparative Fit Index (CFI), Tucker Lewis Index (TLI), Root Mean Square Error of Approximation (RMSEA) and Root Mean Square Residual (RMR)^26,37^. Following the recommendation by Byrne (2013) and Kline (2011), the model was considered to have an adequate fit when the χ2/df ratio was < 5.0, the value of both absolute fit indices (GFI and AGFI) and the comparative fit indices (CFI and TLI) were > 0.90 and both RMSEA and RMR values were < 0.08. Although the chi-square test (χ^2^) is widely used for evaluating model fit, it is highly sensitive to sample size. A significant *p*-value (*p* < 0.05) indicates a discrepancy between the observed and model-implied covariance matrices, suggesting poor model fit. However, this test should not be interpreted in isolation and should be considered alongside other fit indices (e.g., RMSEA, CFI, TLI), which provide a more comprehensive assessment of model adequacy^26,37^.

## Results

### Characteristics of the study’s participants

A total of 217 women with cancer were included in the study. The mean age of the participants was 55.01 ± 12.45 years. The majority were residents of urban areas (50.69%). In terms of religion, 83.41% reported no religion, while 10.6% identified as Buddhist and 5.99% as Catholic. Regarding education level, 48.39% had a college education, followed by 23.96% with a high school education. Occupationally, 41.01% were engaged in intellectual labor, while 39.63% were involved in manual labor. Marital status indicated that 86.18% were married or living as married. A significant portion of participants (72.35%) reported no chronic diseases. The majority were non-smokers (98.16%) and non-drinkers (98.62%). Regarding physical activity, 74.65% engaged in such activities. Additionally, 27.19% reported a family history of cancer. In terms of treatment, the majority underwent chemotherapy (58.06%), followed by surgery (35.02%). The most common method used to monitor and manage symptoms was regular health check-ups (88.48%). More details are displayed in Table 1.

**Table 1:** Demographic and clinical characteristics.

Variables	Categories	n	%
Age (Mean ± SD)		55.01 ± 12.45	
Place of residence	Urban	110	50.69
	Rural/Mountainous	107	49.31
Religion	No	181	83.41
	Buddhism	23	10.6
	Catholic	13	5.99
Education level	Elementary	14	6.45
	Middle School	46	21.2
	High School	52	23.96
	College	105	48.39
Occupation	Not employed	42	19.35
	Blue collar (farmers, vendors, construction workers, laborers, etc.)	86	39.63
	White collar (teachers, healthcare professionals, office workers, military personnel, etc.)	89	41.01
Marital status	Single	11	5.07
	Divorced/Widowed	19	8.76
	Married/Living as married	187	86.18
	Yes	205	94.47
Chronic diseases (e.g., hypertension, diabetes, liver disease, kidney disease)	No	157	72.35
	Yes	60	27.65
Smoking	No	213	98.16
	Yes	4	1.84
Physical activities	No	55	25.35
	Yes	162	74.65
Family history of cancer	No	158	72.81
	Yes	59	27.19
Past/Current treatment therapy	Surgery	76	35.02
	Chemotherapy	126	58.06
	Radiotherapy	41	18.89
	Immunotherapy	5	2.3
	Hormone therapy	30	13.82
	Others	49	22.58
Methods used to monitor and manage symptoms of the disease	No	17	7.83
	Regular health check-ups	192	88.48
	Technological devices (smartphones, smartwatches, etc.)	8	3.69
	Online support (online forums, Facebook groups, etc.)	6	2.76
	Total	217	100

### Prevalence of symptoms among the study’s participants

Table 2 illustrates the prevalence of physical and psychological symptoms among the participants. Fatigue and sleep disturbances were reported most frequently, with fatigue being the most prevalent symptom, affecting 77% and 73% participants, respectively. Other common reported symptoms including pain (65%) and appetite loss (63%).

**Table 2:** Prevalence of symptoms.

N %	Never	Rarely (1 time/week)	Sometimes (1–2 times/week)	Often (3–4 times/week)	Usually (5–6 times/week)	Always	Total
Fatigue	49	47	54	38	17	12	217
	22.58	21.66	24.88	17.51	7.83	5.53	100
Appetite loss	80	41	46	30	9	11	217
	36.87	18.89	21.2	13.82	4.15	5.07	100
Pain	76	58	35	27	11	10	217
	35.02	26.73	16.13	12.44	5.07	4.61	100
Sleep disturbances	57	42	46	33	23	16	217
	26.27	19.35	21.2	15.21	10.6	7.37	100
Hair loss	77	31	32	18	19	40	217
	35.48	14.29	14.75	8.29	8.76	18.43	100
Nausea	113	46	30	14	5	9	217
	52.07	21.2	13.82	6.45	2.3	4.15	100
Sexual issue	130	42	24	6	4	11	217
	59.91	19.35	11.06	2.76	1.84	5.07	100
Mood issue	110	42	26	16	16	7	217
	50.69	19.35	11.98	7.37	7.37	3.23	100

### Frequency of mental health issues among the study participants

Regarding mental health, the majority of participants experienced none or minimal anxiety (83.41%) and none or minimal depression (74.65%). However, mild anxiety (11.52%) and mild depression (17.97%) were also prevalent. A smaller percentage reported moderate to severe levels of anxiety and depression. Additionally, in terms of perceived stress, the majority of participants reported moderate stress (65.44%), while a notable portion reported low stress (34.56%), as presented in Table 3.

**Table 3:** Frequency of Mental Health issues.

		N (n = 217)	Percentage
Anxiety (GAD-7)	None or minimal anxiety	181	83.41
	Mild anxiety	25	11.52
	Moderate anxiety	8	3.69
	Severe anxiety	3	1.38
Depression (PHQ-9)	None or minimal depression	162	74.65
	Mild depression	39	17.97
	Moderate depression	10	4.61
	Moderately severe depression	4	1.84
	Severe depression	2	0.92
Stress (PSS-10)	Low stress	75	34.56
	Moderate stress	142	65.44
	High perceived stress	0	0

### Identifying symptom clusters

#### *Factor loading* > *0.5*

To extract symptom clusters, we utilized Exploratory Factor Analysis (EFA) with principal components and maximum variance rotation method. A total of 11 items were included in the EFA, with an occurrence rate of ≥ 25%. Tests for the suitability of structure detection showed a Kaiser–Meyer–Olkin value of 0.822 and a Bartlett test with *p* < 0.001, indicating the data were suitable for EFA. Cronbach’s α was calculated for each factor to evaluate the internal consistency of the symptom clusters. A Cronbach’s α of 0.7 or higher represents good consistency validity. In this study, variables with factor loadings less than 0.5 were excluded, as statistically, a correlation lower than that would produce too many factors in factor analysis.

A common practice in factor analysis is to retain only those factors with eigenvalues greater than one (Kaiser, 1960; Costello & Osborne, 2005). This criterion suggests that a factor must explain more variance than a single observed variable would on its own. Essentially, an eigenvalue greater than one indicates that the factor accounts for a significant portion of the total variance in the data, justifying its inclusion in the final model. In this study’s analysis, two distinct factors with eigenvalues greater than 1.00 were retained, accounting for 54.66% of the total variance. Factor loadings represent the correlation between observed variables and their underlying factors; thus, higher loadings indicate a stronger relationship. The 0.5 factor loading threshold was selected to ensure that each item contributes meaningfully to its respective factor, enhances the clarity and reliability of the identified symptom clusters, and minimizes cross-loadings (Hair et al., 1998; MacCallum et al., 1999; Stevens, 1992). Factor 1 included fatigue, appetite loss, pain, sleep issues, hair loss, nausea, and sexual issues, with a variance contribution rate of 35.66% and a Cronbach’s α of 0.83, indicating high internal consistency. Factor 2 comprised mood issues, personal stress, depression, and anxiety, with a variance contribution rate of 19.00% and a Cronbach’s α of 0.6102, reflecting moderate reliability.

These findings delineate two main symptom clusters: the *physical cluster* (Factor 1) and the *psychological cluster* (Factor 2). This distinction aids in comprehending the different dimensions of symptom experiences and can inform targeted approaches for treatment or intervention (Table 4).

**Table 4:** Factor analysis of symptom clusters.

Symptom	Factor 1	Factor 2
Fatigue	0.7993	
Appetite loss	0.8139	
Pain	0.6747	
Sleep issue	0.6318	
Hair loss	0.6884	
Nausea	0.7570	
Sexual issue	0.5062	
Mood issue		0.5231
Personal stress		0.5696
Depression		0.7610
Anxiety		0.8331
Varian contribution rate, %	35.66	19.00
Cronbach’s Alpha	0.8345	0.6102

### Factors influencing symptom clusters

Based on the conceptual framework developed from the literature review, the potential socio-demographic and behavioral independent variables included age, place of residence, religion, educational level, occupation, marital status, chronic diseases, smoking status, alcohol consumption, and physical activity. Before conducting the analysis, the assumptions of linear regression (linearity, normal distribution of residuals, homoscedasticity, and no multicollinearity) were examined, and all were met, with VIF < 1.6 and a Durbin–Watson value close to 2 (Table 5).

**Table 5:** Multiple linear regression analysis of the factors on symptom clusters.

	Factor 1 (physical cluster)						Factor 2 (psychological factor)					
Variable	B (SE)	β	t	*p*	Tolerance	VIF	B (SE)	β	t	*p*	Tolerance	VIF
(Constant)	0.425 (1.040)	–	0.409	0.683	–	–	1.257 (1.015)	–	1.238	0.217	–	–
Age	0.00005 (0.007)	0.001	0.008	0.994	0.63	1.587	0.013 (0.007)	0.156	1.898	0.059	0.63	1.587
Place of residence	0.318 (0.156)	0.159	2.037	0.043	0.734	1.363	− 0.155 (0.152)	− 0.078	− 1.021	0.309	0.734	1.363
Religion	0.054 (0.115)	0.033	0.466	0.642	0.893	1.12	0.031 (0.112)	0.019	0.277	0.782	0.893	1.12
Education level	− 0.038 (0.094)	− 0.037	− 0.404	0.687	0.543	1.842	− 0.032 (0.092)	− 0.031	− 0.35	0.727	0.543	1.842
Occupation	0.263 (0.112)	0.197	2.349	0.02	0.639	1.566	− 0.007 (0.109)	− 0.005	− 0.067	0.947	0.639	1.566
Marital status	− 0.245 (0.136)	− 0.124	− 1.797	0.074	0.945	1.058	0.154 (0.133)	0.078	1.158	0.248	0.945	1.058
Chronic diseases	− 0.051 (0.160)	− 0.023	− 0.321	0.749	0.866	1.154	− 0.328 (0.157)	− 0.147	− 2.094	0.037	0.866	1.154
Smoking	− 1.203 (0.613)	− 0.162	− 1.962	0.051	0.655	1.527	− 0.121 (0.599)	− 0.016	− 0.202	0.84	0.655	1.527
Alcohol consumption	0.526 (0.708)	0.062	0.744	0.458	0.652	1.533	− 0.476 (0.691)	− 0.056	− 0.689	0.492	0.652	1.533
Physical activities	0.007 (0.158)	0.003	0.043	0.966	0.945	1.059	− 0.599 (0.154)	− 0.261	− 3.887	< 0.001	0.945	1.059
	R-square = 0.077						R-square = 0.120					

The multivariate regression analysis revealed that Factor 1 was significantly influenced by place of residence (B = 0.318, *p* = 0.043) and occupation (B = 0.263, *p* = 0.020), with marital status (B = − 0.245, *p* = 0.074) showing a marginal effect. Smoking was also approaching significance (B = − 1.203, *p* = 0.051). The R-squared for Factor 1 was 0.077, indicating that the model explains 7.7% of the variance in Factor 1. For Factor 2, physical activity (B = − 0.599, *p* < 0.001) and chronic diseases (B = − 0.328, *p* = 0.037) were significant predictors, while age (B = 0.013, *p* = 0.059) showed a marginally significant effect. The R-squared for Factor 2 was 0.120, indicating that the model accounts for 12% of the variance in Factor 2. Other variables such as education and alcohol consumption did not significantly impact either factor (*p* > 0.05). All VIF values are below the threshold of 5, and Tolerance values are above 0.2, indicating that the predictor variables are not excessively correlated with one another (Table 6).

**Table 6:** Structural Equation Modeling (SEM) for Factor Structure of Symptom Clusters.

	χ2	df	*p*	χ2/df	GFI	AGFI	CFI	TLI	RMSEA	RMR
Model	149.641	43	0.000	3.480	0.896	0.840	0.884	0.852	0.107	0.169

**Fig. 1:**
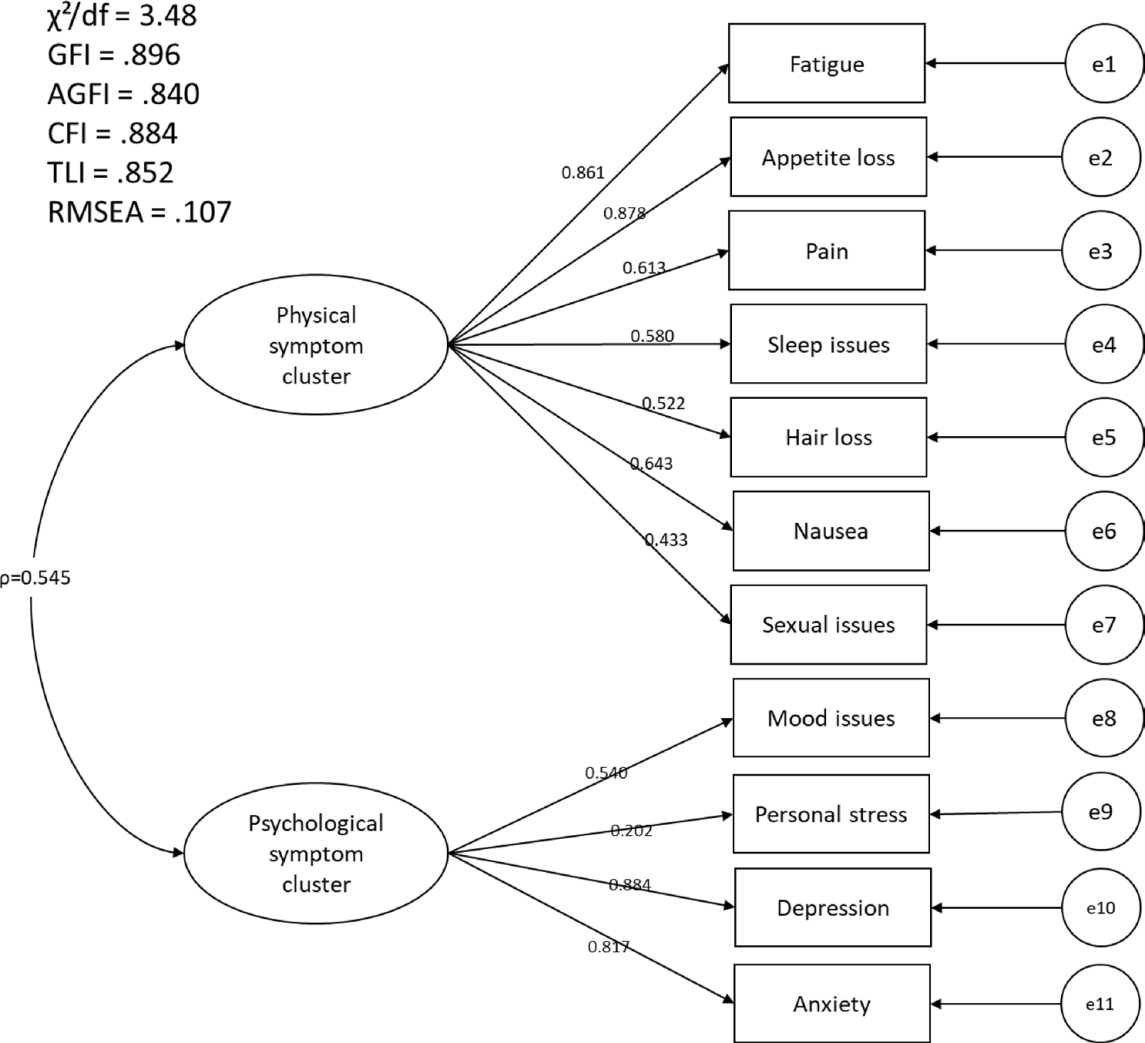
Latent variables (Physical symptom cluster, Psychological symptom cluster) are represented as ovals, and observed symptom indicators are shown as rectangles with standardized factor loadings. Residual variances are indicated by e1 to e11. The double-headed arrow between the two latent variables represents the estimated correlation between clusters (ρ = 0.545). Model fit indices are presented within the figure, χ^2^/df = 3.480, GFI = 0.896, AGFI = 0.840, CFI = 0.884, TLI = 0.852, RMSEA = 0.107 values, which together suggest that although some convergence toward the hypothesized model is observed, the overall fit remains limited, and further validation is warranted.

Structural equation modeling (SEM) showed a moderately acceptable model fit, based on several key indices: χ^2^ = 149.64, df = 43, *p* < 0.001; χ^2^/df = 3.48; GFI = 0.896; AGFI = 0.840; CFI = 0.884; TLI = 0.852; RMSEA = 0.107; RMR = 0.169. Although the chi-square test was statistically significant (*p* < 0.001), which may be due to the large sample size, several other fit indices indicated a moderately acceptable fit. The RMSEA value exceeded the ideal threshold (< 0.08), and the GFI, AGFI, CFI, and TLI were slightly below the commonly accepted cutoff of 0.90. These findings suggest that the model provides only partial support for the hypothesized factor structure, and should be interpreted with caution pending further validation Fig. 1. Structural Equation Model confirming Two-factor structure of Symptom Clusters in Vietnamese women with cancer.

## Discussion

This study revealed that fatigue, appetite loss, and pain were among the most prevalent symptoms experienced by the participants. Fatigue was reported by the majority, with varying degrees of frequency, followed closely by appetite loss and pain. These findings are consistent with existing literature, underscoring the pervasive nature of these symptoms in cancer patients^13,14^.Specifically, the high prevalence of fatigue, reported by the majority of our participants, is consistent with results from a systematic review, the findings of which revealed fatigue-sleep disturbance to be a predominant symptom among breast cancer patients across various treatment stages^38^. This underscores fatigue as a significant burden for cancer patients, necessitating focused interventions. Similarly, our findings regarding appetite loss and pain are in line with previous research. For example, Coleman et al. (2022) identified pain interference as a key component of the SPADE (sleep disturbance, pain interference, anxiety, depression, and energy/fatigue) symptom cluster among cervical cancer survivors^39^. Appetite loss, although not as frequently highlighted as fatigue or pain in some studies, was nonetheless significant in our cohort, reflecting the complex interplay of treatment side effects and overall health status in cancer patients.

In the multivariate regression analysis, the findings show that place of residence and occupation significantly influenced physical symptoms, with urban living and white-collar occupations associated with fewer physical symptoms. Symptom clusters varied with work status including environmental and occupational contexts, highlighting the impact of daily routines and stressors on physical health^40^. For psychological symptoms, physical activity and chronic diseases were significant predictors, with active individuals and those without chronic conditions reporting fewer psychological symptoms. This supports Liska (2020) and Luo et al. (2023), who found a relationship between physical activity and emotional well-being in breast cancer patients and advocates for physical activity as a beneficial intervention for enhancing mental health among cancer survivors^41,42^. Physical activity is known to enhance mood through various mechanisms, such as endorphin release, stress reduction, improved sleep quality, and increased social interaction. Therefore, intervention programs need to focus on incorporating physical activities to improve mental health outcomes for cancer patients^41^. Tailored exercise programs; for example, the Women Wellness Program, Survivorship Wellness Group Program and other interventional programs with regular monitoring and support from healthcare providers are essential for maximizing the mental health benefits of physical activity^43,44^. Interestingly, while age showed a marginally significant effect on both factors, it did not reach statistical significance in this analysis. This finding diverges from studies that have consistently reported age as a significant factor affecting symptom burden in cancer populations^14^. The R-squared values indicate that while these models explain a modest portion of the variance in symptom clusters—7.7% for Factor 1 and 12% for Factor 2, the results still emphasize the complexity of symptom experiences and suggest that additional unmeasured factors may also play a role. Moreover, the absence of significant associations for variables such as education and smoking contrasts with some prior studies that highlighted their relevance in determining symptom severity^12,22^. Our study also did not find a significant association between religion and mental health, which contrasts with previous research indicating that religion can positively affect mental health^5,45–47^. This discrepancy may reflect cultural differences or variations in how these factors interact within different populations.

Through exploratory factor analysis, our study identified two main symptom clusters: physical and psychological. The physical symptom cluster included fatigue, appetite loss, pain, sleep issues, hair loss, nausea, and sexual issues, indicating a high internal consistency. The psychological symptom cluster comprised mood issues, personal stress, depression, and anxiety, reflecting moderate reliability. Our study’s identification of distinct physical and psychological clusters aligns well with the broader literature, which frequently highlights the necessity of addressing both domains for effective symptom management. Luo et al. (2023) further elucidated the multidimensional nature of symptom clusters in their prospective study, identifying gastrointestinal (GI) symptoms, emotional & psychological symptoms, neurological symptoms, menopausal symptoms, and self-image disorder among breast cancer patients^42^. Our study’s clustering of physical symptoms similarly reflects this multidimensionality, albeit focusing on different specific symptoms. The distress caused by the emotional symptom cluster, characterized by worry, difficulty concentrating, and sadness, which aligns closely with our psychological cluster findings^40^. However, our findings differ slightly in that we separated physical and psychological symptoms into distinct clusters, whereas some studies have identified more mixed clusters. For instance, Zhou et al. (2023) identified multiple symptom clusters in cervical cancer patients’ post-radiotherapy/chemotherapy, including combinations of psycho-emotion-related and pain-disturbed sleep-related clusters^48^. This suggests an understanding of symptom interrelationships, which can vary depending on the patient population and cancer type.

Although the model did not fully meet the recommended thresholds for chi-square and RMSEA, the values of other key indices— GFI (0.896), AGFI (0.840), CFI (0.884), and TLI (0.852) —fell within an acceptable range, suggesting a moderate but practically meaningful model fit (Byrne 2013; Hair et al. 2014; Kline 2011). These indices indicate that the proposed two-factor structure provides a reasonable approximation of the symptom clustering phenomenon, particularly within the contextual constraints of a real-world clinical population. The discrepancies between absolute and incremental fit indices may be attributed to sample size limitations or model complexity, both of which are known to affect the sensitivity of fit statistics. Importantly, the model’s ability to capture theoretically consistent groupings of symptoms lends support to its construct validity and its utility for guiding future cluster-based symptom management interventions. Nonetheless, future studies with larger, more diverse samples and inclusion of additional latent variables—such as social support, cancer stage, or health literacy—may enhance model precision and overall fit. Such refinements could further elucidate the multidimensional nature of post-treatment symptomatology and improve the development of tailored survivorship care models.

^41,43–46^This study has several strengths, including the comprehensive sample characteristics and the use of robust statistical methods such as Exploratory Factor Analysis (EFA) and multiple linear regression analysis. These methodologies provided reliable insights into the patterns and determinants of symptom experiences among a diverse sample of women living with and beyond cancer in Vietnam, enhancing the generalizability of the findings. However, the cross-sectional nature of the study limits the ability to infer causality and track symptom changes over time, highlighting the need for longitudinal studies. Potential biases, such as recall bias from self-reported data due to the absence of a validated questionnaire specifically designed for Vietnamese women with cancer, and the limitations of convenience sampling, may also affect the representativeness of the findings. The sample included participants with a range of cancers, allowing to capture a diverse range of symptom experiences. While this may cause heterogeneity in cancer population, this diversity is crucial as it can identify symptom clusters that are common across various cancer types, providing valuable insights into the general patterns of symptom co-occurrence in women with cancer, and be a reference for tailored interventions that consider the unique symptom profiles associated with each cancer type.

Future research should focus on conducting longitudinal studies to monitor symptom progression and clustering over different stages of cancer treatment and survivorship. This will provide deeper insights into the persistence and evolution of symptoms, facilitating more effective and timely interventions. Additionally, exploring a broader range of demographic and clinical factors, such as genetic predispositions and specific cancer types, can help identify high-risk groups and tailor interventions more precisely. Interventional studies targeting the identified symptom clusters could evaluate the efficacy of integrative care models combining pharmacological treatments, lifestyle modifications, technology psychosocial support, and application of technology^49,50^. Complementing quantitative findings with qualitative research can provide rich, contextual insights into the lived experiences of cancer patients, offering a more holistic understanding of symptom management needs and preferences. Addressing these research directions will advance the understanding of symptom experiences and enhance the quality of care for cancer patients, leading to better health outcomes and improved quality of life^51^.

## Conclusion

This study’s identification of distinct physical and psychological symptom clusters among Vietnamese women with cancer provides a foundation for targeted interventions and highlights critical research priorities. The cross-sectional design, while informative, reveals the need for longitudinal investigations to track symptom evolution across treatment phases and survivorship trajectories. Future research should prioritize prospective cohort studies examining how symptom clusters manifest, persist, or resolve over time, enabling the development of predictive models for symptom burden. The modest variance explained by current predictors (7.7–12%) suggests that unexplored factors significantly influence symptom experiences. Investigating genetic predispositions, treatment-specific variables, social support networks, and cultural factors could enhance predictive accuracy and identify high-risk populations. Interventional studies evaluating integrated care approaches—combining pharmacological management, structured physical activity programs, and psychological support—are essential for validating cluster-based treatment strategies.

## Data Availability

The datasets generated and/or analysed during the current study are not publicly available due to privacy and ethical restrictions but are available from the corresponding author on reasonable request.
